# Validity and Reliability Study of Bahasa Malaysia Version of Sino-Nasal Outcome Test 22 in Chronic Rhinosinusitis Patient

**DOI:** 10.21315/mjms2022.29.2.12

**Published:** 2022-04-21

**Authors:** Ramli RAMIZA RAMZA, Zulkifli SHIFA, Abdullah BAHARUDIN, Mohamad SAKINAH, Md Shukri NORASNIEDA

**Affiliations:** Department of Otorhinolaryngology, Head and Neck, School of Medical Sciences, Universiti Sains Malaysia, Kelantan, Malaysia

**Keywords:** sinusitis, rhinitis, SNOT-22, validation study, Bahasa Malaysia version

## Abstract

**Background:**

Chronic rhinosinusitis (CRS) affects 14% of the general population. The Sino-Nasal Outcome Test 22 (SNOT-22) is a reliable instrument assessing the impact of CRS. This study aimed to examine the reliability and construct validity of the translated Bahasa Malaysia version of SNOT-22.

**Methods:**

This cross-sectional study was conducted in the Otorhinolaryngology clinic in Universiti Sains Malaysia (USM). Seventy CRS respondents and 39 healthy participants were included. The Bahasa Malaysia translated SNOT-22 (bmSNOT-22) was produced using rigorous forward and backward translation. Statistical analyses used included feasibility, Cronbach’s alpha, intraclass correlation coefficient, Pearson’s correlation coefficient and factor analysis.

**Results:**

The feasibility was 97.2% in the initial test and 100% in the retest. The Cronbach’s alpha was 0.89 in the initial test. The average intraclass correlation coefficient (ICC) was 0.90, indicating good test-retest reliability. The bmSNOT-22 discriminated between the control group and patients (*t* = 15.33; *P* < 0.001).

**Conclusion:**

The bmSNOT-22 is reliable, and validity established therefore recommended for Malaysia’s clinicians and researchers as a measurement tool for the outcome in sino-nasal disorders such as rhinosinusitis and nasal polyps.

## Introduction

Rhinosinusitis is an inflammation of the sinuses and the nasal cavity; therefore, it is the preferred term to be used rather than sinusitis. As for rhinitis, i.e. inflammation of nasal passage, it can co-exist with sinusitis. The term rhinosinusitis has been used since 1990 and is well adopted globally (1–5). The presentation may have been perceived differently depending on the patient’s initial assessment and treatment. During primary care, the patient is more likely to be diagnosed with rhinitis (6). The same patient presenting at a tertiary centre might be diagnosed with rhinosinusitis. According to the European Position Paper on Rhinosinusitis and Nasal Polyps (EPOS) 2020, the clinical definition of adult rhinosinusitis and paediatric rhinosinusitis are the same except for smell disturbances, i.e. loss or reduction of smell, which can be present or absent in an adult but not as a criterion in children. The children’s clinical definition for rhinosinusitis replaced the smell disturbances with the presence or absence of cough (1). Additional to the symptoms, examination and radiological findings must also be supportive of rhinosinusitis. If the presentation is < 12 weeks, it is defined as acute rhinosinusitis, and if it is ≥ 12 weeks, it is defined as chronic rhinosinusitis (CRS).

CRS is a widespread global health disorder, increasing in both incidence and prevalence. It is not a life-threatening disorder. Nevertheless, it has been shown to significantly impact the quality of life (QOL) demonstrated by Hopkins et al. (7), using a general questionnaire, i.e. a 36-item short form health survey (SF-36). Although an SF-36 demonstrated to be useful in assessing CRS patient’s QOL, a disease-specific questionnaire may be more suitable to evaluate many aspects of the disease (8). Sino-Nasal Outcome Test-22 (SNOT-22) is a widely used disease-specific questionnaire for CRS. It contains 22 questions of CRS-related symptoms/QOL and has been demonstrated for its validity and reliability (9). It has been translated and revalidated from the original English version into several other languages, including Chinese, Portuguese, Greek, French and Thai (8, 10–13).

This study aims to translate and validate SNOT-22 into the Bahasa Malaysia version of SNOT-22 (bmSNOT-22) and to test its reliability in the studied population. A bmSNOT-22 version will be crucially important in helping to establish the desired outcome and improving treatment and predicting the postsurgical improvement in patients with CRS in Malaysia. Research wise, the opportunity to expand a nationwide study dwelling into the QOL of CRS patients using the bmSNOT-22 will provide a wide variety of data to analyse not only in Malaysia but also to the surrounding country that uses the Malay language as their basis of communications, i.e. south Thailand, Brunei and Indonesia.

## Methods

Each participant signed informed consent before inclusion into the study. Permission to use and translate the SNOT-22 was obtained from Piccirillo JF (14). First, SNOT-22 was translated into Bahasa Malaysia language independently by two native Bahasa Malaysia speakers, a doctor experienced in the management of CRS and a professional translator, respectively, who were bilingual in Bahasa Malaysia and English. It was then translated back to English and was assessed for equivalence with the original English version. The second draft was then tested on eight volunteer subjects and reviewed to establish the content validity by a medical expert in rhinology.

This cross-sectional study was done in Otorhinolaryngology, Head and Neck (ORL-Head and Neck), and Rhinology Clinic in Hospital Universiti Sains Malaysia. Its psychometric properties were assessed against the validated Bahasa Malaysia Version of SF-36 acknowledged as the standard gold instrument. All subjects were between 15 years old–75 years old and could read and write Bahasa Malaysia. The control subjects were healthy Malaysian medical students and health personnel of the School of Medical Sciences from Hospital Universiti Sains Malaysia. The CRS group was diagnosed based on the EPOS 2012 (15). Subjects were required to fill up the questionnaire during a clinic visit at presentation and approximately 2 weeks to 1 month later to assess QOL on the generic Bahasa Malaysia version of SF-36 and the bmSNOT-22 questionnaires.

The sample size for this study was calculated using subject-to-observed variable-ratio *N*:*P*; 3:1, meaning that three subjects per item. Since the SNOT-22 study has 22 items, thus the sample size required is 66 subjects (22 × 3 subjects per item). With an addition of a 10% non-response rate, the sample size required was 72 subjects. As for the sample size determination for reliability, STATA 11.0 software was used to determine its internal consistency. The expected Cronbach’s alpha value that was decided was 0.80; therefore, the minimum sample size required within the 95% lower bound confidence interval of alpha 0.700 is 29 subjects.

The sample size of test-retest reliability was also calculated using STATA 11.0 software using alpha at 0.05 and the power of the study at 0.80, the estimated intraclass correlation coefficient (ICC) was at 0.80. Therefore, the calculated sample size for test-retest reliability is 46 subjects. In addition to that value, another 10% non-response rate was added, giving the sample size requirement of 50 subjects.

Statistical analysis was performed using SPSS programme version 20.0 for Windows (SPSS Inc., Chicago, IL, USA). Test-retest reliability was calculated by comparing bmSNOT-22 between the first and second visit, using the ICC. The construct validity was tested using confirmatory factor analysis conducted through extracting factors by principal components, followed by Varimax rotation with Kaiser normalisation. Several factors were set to four to determine the number of rotation factors.

## Results

Seventy CRS patients and 39 healthy controls participated in this study (response rate 97.2%) with an average age of 37.77 years old and 39.49 years old, respectively. The descriptive statistics of each item and scale of bmSNOT-22 and SF-36, including their means, standard deviation (SD), ceiling and floor effects, were evaluated and summated rating scales as shown in Table 1 and Table 2.

As suggested by the Likert scaling criteria, item means of bmSNOT-22 were all roughly equivalent. The item means, and SD of all the items did not differ (range of mean from 1.10 to 2.64 and SD ranged from 0.933 to 1.486) (Table 1). Thus, fulfilling the Likert scaling criteria requirement. The mean bmSNOT-22 sum score was 46.00 (range 0–110) in the initial test and 41.45 (range 0–110) in the retest. The mean difference in the sum score was 5.55 (*t* = 5.288; SD = 7.198; *P* < 0.001). There was a significant difference between the means of the two tests. The mean sum score of bmSNOT-22 was analysed into four respective domains and the findings were summarised in Table 2.

The floor effects are present from questions 16 to 21 of the bmSNOT-22, thus indicating limited responsiveness of these questions when evaluated as a single construct. In this study, floor effects or ceiling effects are considered to be present when it is 30% or more. Therefore, when bmSNOT-22 is evaluated as a single construct, floor effects are observed in the Bahasa Malaysia translated items of ‘sad’ (Q19) and ‘embarrassed’ (Q20), but there were no ceiling effects seen. However, the floor effects were diminished when the bmSNOT-22 was analysed as four different subdomains (Table 2). Pynnonen et al. (16) and Browne et al. (17) divided SNOT-20 into four subdomains, the items ‘cough’ (Q4) and ‘waking up tired’ (Q14) were excluded since it does not load clearly on any construct.

We chose the same four scales in this study but included the two extra items under rhinological subdomains and excluded Q4 and Q14 in the analysis. The four subscales were rhinological symptoms (Q1–Q3, Q5–Q6 and Q21–Q22), ear and facial symptoms (Q7–Q10), sleep function (Q11–Q13), and psychological function (Q15–Q20) (Table 2). Of all the subdomains within SNOT-22, 29.7% of the respondents scored the lowest possible value in the psychological issues domain. There were no ceiling and floor effects present in all the subdomains of bmSNOT-22.

Even in our comparison questionnaire of Bahasa Malaysia version of SF-36, there is a prominent ceiling effect, especially within the role physical (RP) and role emotional (RE) domains that are 54.286% and 40%, respectively. Compared to these two instruments, the Bahasa Malaysia version of SF-36 recorded more patients with minimum scores (floor) and maximum scores (ceiling).

### Reliability

The scores for each item in the initial test of bmSNOT-22 are shown in Table 3. The Cronbach’s alpha scores for the bmSNOT-22 were 0.89 (above 0.7) in the initial test for the CRS patients, indicating high internal consistency. The Cronbach’s alpha in the retest was 0.90. Both values suggest good internal consistency within bmSNOT-22. Table 3 showed that good reliability is achieved in all 22 items of this questionnaire, as proven with lower Cronbach’s alpha values.

The ICC for each item of the bmSNOT-22 was evaluated for test-retest reliability and presented in Table 4. The ICC for single measures is 0.830 at 95% confidence interval (CI) (0.725, 0.897). The CI width for single measures was 0.172. The average measures of ICC were 0.907 at 95% CI between 0.841 and 0.946, and its width is narrow was 0.105. Both of these values indicate the high reliability of repeated measures.

### Validity

Both bmSNOT-22 and the Bahasa Malaysia version of SF-36 were examined against healthy controls. From the analysis, the bmSNOT-22 discriminated between patients known to suffer from CRS and the control group. Mean scores between CRS patients and the control group are shown in Table 5.

The mean difference in the sum score was 36.69. It demonstrates statistically significant variability between these groups (*P* < 0.001; *t* = 15.33). Thus, there was a significant difference between the means of these two groups. In contrast, the Bahasa Malaysia version SF-36 was also examined against the healthy controls (Table 5), and statistically, it showed no significant difference between these two groups (*P* = 0.70; *t* = 0.39).

Kaiser-Meyer-Olkin (KMO) of sampling adequacy measured 0.731 (> 0.6) and Bartlett’s test of sphericity is significant (*P* < 0.0001). Thus, it enables factor analysis to be carried out in this study. Factor analysis was carried out by extracting factors through principal component analysis followed by rotation methods using Varimax rotation with Kaiser normalisation.

**Figure 1:**
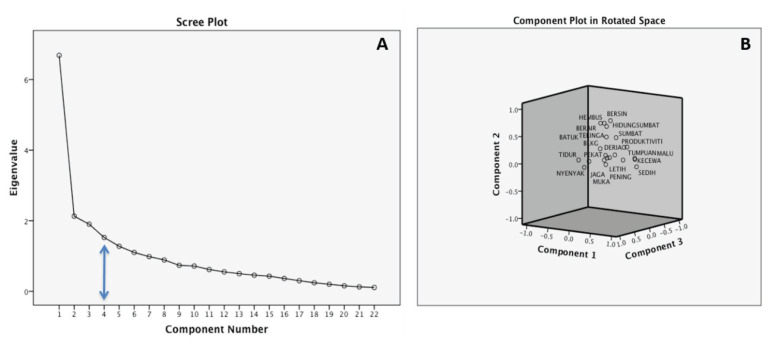
A) Scree plot for bmSNOT-22, showing the cut point (blue arrow) at which, the slope appears to change into minor decrement suggesting four factors. B) Component plot in rotated space

The unforced factor analysis with Varimax rotation and principal component analysis on the bmSNOT-22 produced six factors with Eigenvalues greater than 1. The six factors captured 66.5% of the total variance. However, based on the screen plot (graph of the percentage of variance explained) (Figure 1), the cut point at which the slope appears to change into minor decrement suggested four factors. The decision on the number of subdomains was made in this study after confirmatory factor analysis from the actual questionnaire design that consists of four subdomains (16–17).

Question loading into separate subdomains was calculated using a rotated factor matrix. Pure variable only has loading > ± 0.3 on only one factor. From the result of the four-factor analysis, we were able to determine that ‘*deria rasa atau bau*’ (Q21), ‘*lelehan pasca-hidung*’ (Q5) and ‘*hingus yang pekat*’ (Q6) have the highest load, not within the expected subdomains. For example, we would have expected to have Q6 to load into rhinological symptoms. Still, it is categorised under the ear or facial symptoms and Q21 together with Q5 load almost equally on more than one construct (Table 6).

The other questions have the highest load into the expected subdomains. The items of ‘*sedih*’ (Q19), ‘*kecewa/resah/rasa mudah marah*’ (Q18), ‘*keletihan di siang hari*’ (Q15), ‘*penumpuan berkurangan*’ (Q17), ‘*produktiviti berkurangan*’ (Q16), ‘*malu*’ (Q20) and ‘*berasa letih ketika bangun*’ (Q14) load into psychological function. Rhinological symptoms covers the items were ‘*bersin’* (Q2), ‘*perlu menghembus hidung*’ (Q1), ‘*hidung berair*’ (Q3), ‘*hidung tersumbat’* (Q22) and ‘*batuk’* (Q4). ‘*Sakit bahagian muka atau tekanan pada muka*’ (Q10), ‘*sakit telinga/tekanan*’ (Q9), ‘*telinga terasa mampat/tersumbat telinga*’ (Q7) and ‘pening’ (Q8) were categorised under ear or facial symptoms. Sleep function contains three questions: ‘*terjaga di waktu malam*’ (Q12), ‘*kekurangan tidur nyenyak waktu malam*’ (Q13), ‘*susah untuk tidur*’ (Q11) (Figure 1). Therefore, from this analysis, it can be concluded that there is existence of four unique construct within the bmSNOT-22 that is the rhinological symptoms, ear or facial symptoms, psychological and sleep functions which we believe might improve the effectiveness of this questionnaire in clinical management.

## Discussion

In many descriptive studies, CRS has a greater impact on BP and social functioning than angina, congestive heart failure and chronic obstructive pulmonary disease (7). The importance of patient-reported outcome measures (PROM) has gained much attention in clinical studies and quality of care. The impact of this disorder and its treatment on QOL should be measured directly.

The SNOT-22 is a further modification of SNOT-20 where two additional items were measured: i) nasal blockage and ii) loss of sense of taste and smell. The scoring system has been simplified by removing the importance rating. The importance rating is added initially to improve its content validity (i.e. the ability of the instruments to measure all important aspects of the related disease adequately) (7). SNOT-22 has been validated in Chinese, Danish, Czech and Thai versions. The Bahasa Malaysia translated SNOT-22 is a 22-item instrument used to measure the QOL among CRS patients in the Bahasa Malaysia speaking population.

### Descriptive Data

In this study, a total of 70 participants willingly consented to this study, and 55 of them were examined again for the test-retest reliability between 2 weeks and 4 weeks later. All the participants need to fulfil the inclusion and exclusion criteria. The consented patients were recruited from convenience sampling taken from rhinology and general ORL-HNS clinic HUSM. The period taken for this study is 7 months, starting from December 2011 until June 2012. The age distribution among our study participants ranged from 18 years old to 72 years old, with a mean of 37.77 years old. In contrast to Pynnonen et al. (16) and Browne et al. (17), which shows mean age of the subjects are 46.6 years old and 48 years old, respectively. The highest number of participants was between 26 years old and 35 years old (32.90%), followed by 36 years old and 45 years old (21.40%). Almost 54.3% of participants were aged between 26 years old and 45 years old. This is because younger patients are more willing to participate in this study than the elderly, which is troublesome to fill in the form because of either vision or illiteracy problems. Similar to other studies, the majority were female participants, 55.7% (*n* = 39) and males were 44.3% (*n* = 31) (16–17). A vast majority of participants included in this study were 82.9% (*n* = 58) Malay, 12.9% (*n* = 9) Chinese and 4.3% (*n* = 3) from other ethnic background. However, these figures do not reflect the actual multi-racial variation as the predominant Kelantan population is the Malay ethnicity which accounts for 90%–95%. None of the Indian ethnic population was included in this study due to the scarce Indian ethnicity in the Kelantan population.

### Likert Scaling Assumptions

SNOT-22 and SF-36 scores use the Likert scales assumptions method. It has been widely used for scale construction because of its simplicity and accurately yields reliable scores. In this study, Likert scale assumptions and other necessary assumptions have been determined. The bmSNOT-22 showed equal variances and equal total score variability. Thus, Likert scale assumptions are met. All the items’ means were roughly equivalent in this study and avoiding item standardisation. All the items’ mean scores ranged from 1.10 to 2.64, and standard deviations ranged from 0.933 to 1.486.

### Missing Values and Feasibility

A high percentage of missing data may indicate problems with the translation process and respondents’ confusion on completing that part of the questionnaire (18). Therefore, it poses serious problems, especially in a self-administered questionnaire. To minimise problems with missing data, an interviewer administration of the questionnaire is preferred. Luckily, there was no missing data in this study despite both were self-administered questionnaires. This indicates that the translation process of the instrument was well understood by respondents and seen as relevant.

The feasibility of an instrument in measuring health related QOL (HRQOL) is determined by examining the floor effect, which is the number of respondents with the lowest possible score, and the ceiling effect, which indicates the highest possible score (19–20). Table 1 and Table 2 showed the floor and ceiling effect of each item on the bmSNOT-22. Osborne et al. (21) stated that the low percentage indicates substantial spread across the entire range of scale within an item and too high at either end of the scales defines that the respondents are too ill or too well. The ceiling and floor effect problems are that the items and scales will have poor discrimination. Thus, sensitivity and responsiveness will be reduced (18–19). In this study, floor or ceiling effects are considered to present for each item at 30% or more. Floor effects are observed in the Bahasa Malaysia translated items of ‘sad’ (Q19) and ‘embarrassed’ (Q20), but there were no ceiling effects seen. According to Terwee et al. (19), floor and ceiling effects were present if more than 15% of respondents achieved the lowest or highest score. The Q1–Q15 and Q22 had no ceiling or floor effects by using values less than 15%. However, when all the items of bmSNOT-22 were divided into subdomains, there were no floor and ceiling observed, which indicates that bmSNOT-22 is a good and responsive questionnaire, similar to studies by Pynnonen et al. (16) on SNOT-20.

### Reliability Assessment

Assessment of reliability and validity of a questionnaire design is part of establishing a sound psychometrics evaluation (21). Reliability refers to consistency, dependability and stability. In this study, two types of reliability are assessed that is the internal consistency and test-retest reliability. Internal consistency, measured using Cronbach’s alpha, scores the initial test 0.89 and 0.9 in the retest indicating high internal consistency. These findings were almost similar to another form of a translated version of SNOT-22.

Another form of reliability is stability or repeatability over time that is the test-retest reliability. It is assessed by administering the questionnaire to respondents on two different occasions and examining the correlation between scores. Test-retest reliability correlations for summary scores should be greater than 0.7. It is obtained by correlating the matched responses in the initial and subsequent repeat questionnaires. In this study, the average intraclass correlation coefficient for the bmSNOT-22 is 0.907 at 95% CI. It ranges between 0.841 and 0.946. The narrow width value of 0.105 proved that the bmSNOT-22 has a high test-retest reliability of repeated measures.

### Validity Assessment

Validity refers to the appropriateness, meaningfulness and usefulness of the specific inferences made from the total scores. There are various forms of validity. In this study, we construct validity for the bmSNOT-22 using face validity, content, construct and criterion validity. We conducted respondent testing on eight CRS patients and three medical doctors to establish face validity, and the questionnaire appeared appropriate to them, and the bmSNOT-22 was fulfilled.

In addition, to construct the validity of an instrument, factor analysis needs to be carried out. In the previous studies in English and Czech versions of SNOT-22, no attempt was made to investigate the grouping of the items in SNOT-22. Studies were done by Pynnonen et al. (16) and Browne et al. (17) to validate the subdomains in SNOT-20. Their studies concluded that items of ‘cough’ and ‘waking up from sleep’ did not load clearly on any construct. Pynnonen et al. (16) added the item ‘fatigue’ to this list. However, both of these studies concluded that it is methodologically sound and clinically meaningful by dividing SNOT-20 into four subscales for rhinological symptoms, ear/facial symptoms, sleep function and psychological function domains.

We construct the factor analysis in this study by extracting factors through principal component analysis, followed by rotation methods using Varimax rotation with Kaiser normalisation. Prior to any factor analysis, sampling adequacy using KMO and significant Bartlett’s test of sphericity were demonstrated. By using exploratory factor analysis with Eigenvalues > 1, six components were produced. However, the scree plot suggested four factors, and based on the original SNOT-22 questionnaire, four subdomains were decided to be used in this study. Based on the study by Pynnonen et al. (16) and Browne et al. 17), the analysis was constructed using the impact of psychological function, sleep function, rhinological symptoms, and ear or facial symptoms. The result supported the existence of more than one unique construct within the bmSNOT-22. The construct that we discovered in our analysis fit with our clinical understanding of sino-nasal disease, but there are areas of uncertainty. We would expect that the items ‘*lelehan pasca-hidung*’ (Q5) and ‘*deria rasa atau bau*’ (Q21) would load purely into rhinological symptoms, but it loads almost equally onto more than one construct with the highest values within the ear or facial symptom (Q5) and psychological functions (Q21). Question on ‘*hingus yang pekat*’ (Q6) load purely onto an ear or facial symptom. The other 19 questions have the highest values within the expected subdomains. Therefore, we believe that the usage of bmSNOT-22 in clinical research may help to improve the quality of data produced for clinical management strategies.

As part of criterion validity fulfilment, discriminative validity was also conducted. We hypothesised that bmSNOT-22 could discriminate between patients known to suffer from CRS and a group of healthy individuals. The mean total score of this bmSNOT-22 is 46.00 in CRS patients and 9.31 in healthy individuals. This result agrees with the mean sum score of Czech and English versions SNOT-22. Both studies reported the score among CRS patients was 38.52, 35.7–44.8, respectively, and in healthy controls were 10.22, 9.3. An independent *t*-test was carried out to measure known group validity in detecting expected differences across groups and a *P*-value of 0.05 or less is considered significant. The results showed *t* = 15.33; *P* < 0.001, and the hypothesis is met (Table 5). This highlights the significant and statistically different variability between these two groups.

To further support the importance of bmSNOT-22 usage in sino-nasal disease, the Bahasa Malaysia version SF-36 was also examined against the healthy controls. The mean score was 55.32 in CRS patients and 54.34 within the non-sino-nasal population. There was no significant difference between these two groups (*P* = 0.70; *t* = 0.39) (Table 5).

The concurrent validity of QOL instruments is a measure of agreement between the results obtained from the survey instruments and other instruments acknowledged as the ‘gold standard’ within the same targeted population. The establishment of concurrent validity is part of criterion validity. The validity of the bmSNOT-22 was assessed against the Bahasa Malaysia version SF-36 using a correlation coefficient. In this study, we used the Pearson correlation coefficient (*r*) to examine the correlation between corresponding domains of the bmSNOT-22 and Bahasa Malaysia version SF-36. It was hypothesised that there should be inter-domain correlations between bmSNOT-22 and Bahasa Malaysia version of SF-36.

The correlation coefficient always lies between −1 and +1. It indicates a perfect positive or negative linear relationship between two variables. Negative correlations were observed between all subdomains within the bmSNOT-22 and RE component of Bahasa Malaysia version SF-36. This means that when an item increases, the second variable will decrease. The majority of the subscales within the bmSNOT-22 were weakly correlated with the eight subscales of the Bahasa Malaysia version of SF-36. However, the subscale of RP in Bahasa Malaysia version SF-36 statistically correlates significantly with ear or facial symptoms (*r* = −0.259; *P* < 0.05) and psychological issues (*r* = −0.304; *P* < 0.01) in bmSNOT-22. BP domains in Bahasa Malaysia version SF-36 were also statistically significant with psychological issues (*r* = 0.498; *P* < 0.01) in bmSNOT-22.

From the analysis done between the bmSNOT-22 scores and the Bahasa Malaysia version SF-36, the correlation was significant (*P* < 0.05) for two out of eight dimensions: the RP and BP subdomains. The significant correlations between two out of eight dimensions in Bahasa Malaysia version SF-36 and the weak correlations obtained in this present study may have been due to the small sample size (22).

However, despite the weak correlation, its validity can be established by fulfilling content, construct and discriminant validity. Therefore, bmSNOT-22 is a reliable, valid and sensitive tool. Using a larger sample size can determine its psychometric properties more precisely with a plan to validate further within various clinical practices.

Lastly, we have ensured that unlike its predecessor, the SNOT-20, the SNOT-22 has the most disease-specific items. We have demonstrated in this study that healthy individuals report significantly lower scores on the SNOT-22. However, patients with other medical conditions may also report some of the symptoms included in the instruments. It is important to note that this instrument should not be used for diagnostic purposes, but it is developed to capture the impact a given disease has on all QOL aspects.

## Conclusion

The bmSNOT-22 is good for health status measurement in comparison to the standard generic health status questionnaire. All the items within bmSNOT-22 had high communalities. Therefore, it is established that the items share variance, and a good correlation exists between items within the bmSNOT-22. In conclusion, this study has found that the bmSNOT-22 was a valid and easy-to-use instrument. It can be used to facilitate clinical practice and research purposes and to highlight the impact of CRS on patients’ QOL.

## Figures and Tables

**Table 1:** Item descriptive statistics of the bmSNOT-22 in CRS patients (*n* = 70)

Item	Mean	SD	% Floor (*n*)^a^	% Ceiling (*n*)^b^
Q1	2.370	0.980	0.000 (0)	0.000 (0)
Q2	2.340	1.048	0.000 (0)	0.000 (0)
Q3	2.370	1.079	0.000 (0)	0.000 (0)
Q4	2.190	1.067	0.000 (0)	0.000 (0)
Q5	2.530	1.100	0.000 (0)	0.000 (0)
Q6	2.240	1.069	0.000 (0)	0.000 (0)
Q7	2.010	0.951	0.000 (0)	0.000 (0)
Q8	2.460	1.138	0.000 (0)	0.000 (0)
Q9	2.000	0.933	0.000 (0)	0.000 (0)
Q10	2.360	1.064	0.000 (0)	0.000 (0)
Q11	2.290	1.131	0.000 (0)	0.000 (0)
Q12	2.160	0.987	0.000 (0)	0.000 (0)
Q13	2.170	1.021	0.000 (0)	0.000 (0)
Q14	2.270	1.102	0.000 (0)	0.000 (0)
Q15	2.460	1.003	0.000(0)	0.000 (0)
Q16	1.640	1.341	24.800 (17)	4.286 (3)
Q17	1.570	1.389	28.571 (20)	2.857 (2)
Q18	1.740	1.421	27.143 (19)	4.286 (3)
Q19	1.190	1.171	**32.857 (23)**	1.429 (1)
Q20	1.100	1.264	**35.714 (25)**	1.429 (1)
Q21	1.900	1.486	24.296 (17)	2.857 (2)
Q22	2.640	1.485	4.286 (3)	5.714 (4)

Notes:

a Percentage of patients scoring lowest possible value;

b Percentage of patients scoring highest possible value

**Table 2:** Descriptive statistic for bmSNOT-22 according to subdomain and Bahasa Malaysia version SF-36 scores among CRS patients

	Mean sum score (SD) (*n* = 70)	Range	%Floor	%Ceiling
bmSNOT-22				
A. Rhinological symptoms	16.40 (5.281)	0 35	14.280	4.285
B. Ear/facial symptoms	8.828 (2.884)	0 20	0.000	0.000
C. Sleep function	6.614 (2.622)	0 15	0.000	0.000
D. Psychological function	9.70 (6.094)	0 30	29.714	2.857
SF-36				
A. Physical Functioning	69.9286 (24.620)	10 100	1.429	8.571
B. RP	61.7857 (38.477)	0 100	14.286	40
C. BP	36.9841 (19.358)	0 100	11.429	0
D. General Health	48.8571 (10.570)	15 100	0	0
E. Vitality	49.8571 (11.260)	0 85	0	0
F. Social Functioning	45.0000 (11.925)	13 100	0	0
G. RE	75.7143 (93.237)	0 100	20	54.286
H. Mental Health	54.4000 (11.178	16 100	0	0

Notes: The bmSNOT-22 scores from 0 to 35 with total scores 110, SF-36 score from 0 to 100;

* Question on cough (Q4) and wake up tired (Q14) were e xcluded, as it does not load clearly on any construct (16, 17)

**Table 3:** Internal consistency for the bmSNOT-22 (*n* = 70)

bmSNOT-22 items	No. of item	Corrected item-total correlation	Cronbach’s alpha if item deleted
Q1	1	0.582	0.884
Q2	1	0.612	0.886
Q3	1	0.514	0.883
Q4	1	0.432	0.883
Q5	1	0.480	0.887
Q6	1	0.428	0.886
Q7	1	0.500	0.886
Q8	1	0.458	0.884
Q9	1	0.480	0.885
Q10	1	0.425	0.884
Q11	1	0.556	0.886
Q12	1	0.687	0.886
Q13	1	0.563	0.886
Q14	1	0.509	0.884
Q15	1	0.618	0.884
Q16	1	0.724	0.875
Q17	1	0.753	0.877
Q18	1	0.728	0.878
Q19	1	0.802	0.880
Q20	1	0.729	0.879
Q21	1	0.542	0.880
Q22	1	0.589	0.884

**Table 4:** Test-retest reliability of the bmSNOT-22 (*n* = 55)

Measures	ICC	95% CI	

		Lower bound	Upper bound
Single	0.830	0.725	0.897
Average	0.907	0.841	0.946

**Table 5:** The bmSNOT-22 sum scores between CRS patients and healthy control

	Subjects	*n*	Mean sum score (SD)	*t* (df)^a^	95% CI		*P*-value

					Lower	Upper	
bmSNOT-22	Healthy	39	9.31 (7.21)	15.33 (107)	41.44	31.95	< 0.001
	CRS	70	46.00 (13.92)				
SF-36	Healthy	39	54.34 (5.77)	0.389 (107)	5.93	3.98	0.70
	CRS	70	55.32 (14.97)				

Note:

a Degree of freedom

**Table 6:** Question loadings for bmSNOT-22 constructs (*n* = 70)

bmSNOT-22 items	Psychological function	Rhinology symptom	Ear/facial symptom	Sleep function
*Sedih* (Q19)	**0.877**			
*Kecewa/resah/rasa mudah marah* (Q18)	**0.809**			
*Keletihan di siang hari* (Q15)	**0.714**			
*Penumpuan berkurangan* (Q17)	**0.700**		0.339	
*Produktiviti berkurangan* (Q16)	**0.670**	0.366		
*Malu* (Q20)	**0.662**		0.356	
*Berasa letih ketika bangun* (Q14)	**0.636**			
*Deria rasa/bau* (Q21)	0.427	0.401		
*Bersin* (Q2)		**0.808**		
*Perlu menghembus hidung* (Q1)		**0.711**		
*Hidung tersumbat* (Q22)		**0.633**		
*Hidung berair* (Q3)		**0.682**		
*Batuk* (Q4)	0.332	**0.484**		
*Sakit bahagian muka/tekanan pada muka* (Q10)			**0.729**	
*Sakit telinga/tekanan* (Q9)			**0.709**	
*Telinga terasa mampat/tersumbat telinga* (Q7)		0.398	**0.530**	
*Pening* (Q8)	0.358		**0.519**	
*Hingus yang pekat* (Q6)			0.477	
*Lelehan pasca-hidung (hingus mengalir di bahagian belakang hidung/tekak* (Q5)		0.372	0.418	0.330
*Terjaga di waktu malam* (Q12)				**0.853**
*Kekurangan tidur nyenyak waktu malam* (Q13)				**0.770**
*Susah untuk tidur* (Q11)				**0.745**

Notes: Values lower than 0.3 are suppressed; The bolded values have the highest values within the expected subdomains
